# Exploring the Link Between Renal Function Fluctuations Within the Physiological Range and Serum/CSF Levels of NfL, GFAP, tTAU, and UCHL1

**DOI:** 10.3390/ijms26020748

**Published:** 2025-01-17

**Authors:** Kimberly Koerbel, Yavor Yalachkov, Tabea Rotter, Martin A. Schaller-Paule, Jan Hendrik Schaefer, Lucie Friedauer, Jasmin Jakob, Falk Steffen, Stefan Bittner, Christian Foerch, Michelle Maiworm

**Affiliations:** 1 Department of Neurology, University Hospital Frankfurt, Goethe University, 60590 Frankfurt am Main, Germany; 2 Practice for Neurology and Psychiatry Eltville, 65343 Eltville am Rhein, Germany; 3 Department of Neurology, Focus Program Translational Neuroscience (FTN) and Immunotherapy (FZI), Rhine Main Neuroscience Network (Rmn2), Medical Center of the Johannes Gutenberg University Mainz, 55131 Mainz, Germany; 4 Department of Neurology, Klinikum Ludwigsburg, 71640 Ludwigsburg, Germany

**Keywords:** kidney function, biomarkers, NfL, serum, cerebrospinal fluid

## Abstract

Impaired renal function can influence biomarker levels through mechanisms involving blood–brain barrier integrity and clearance pathways; however, the impact of variations within normal renal function remains unclear. The main aim of this study was to determine whether adjustment for the specific level of renal function is necessary when renal function remains within physiological levels. We studied n = 183 patients (NID n = 122; other neurological diseases n = 39; somatoform controls n = 22) who underwent lumbar puncture at University Hospital Frankfurt. Serum and cerebrospinal fluid (CSF) levels of neurofilament light chain (NfL), glial fibrillary acidic protein (GFAP), total tau protein (tTAU), and ubiquitin C-terminal hydrolase-L1 (UCHL1) were measured using the single molecule array (SIMOA) technique. Estimated glomerular filtration rate (eGFR) correlated negatively with CSF GFAP (r = −0.217, *p* = 0.004) and serum NfL (r = −0.164, *p* = 0.032). Patients with impaired renal function exhibited higher CSF NfL (*p* = 0.036) and CSF GFAP (*p* = 0.026) levels. However, these findings did not remain significant after adjusting for BMI and age. Importantly, in patients with normal renal function, no significant correlations with eGFR and biomarker levels were observed after adjustment. Our findings indicate that serum and CSF concentrations of NfL, GFAP, tTAU, and UCHL1 are not significantly affected by fluctuations in physiological kidney function but emphasize the importance of considering comorbidities in impaired renal function when interpreting biomarker levels.

## 1. Introduction

In recent years, fluid biomarkers have received increasing attention in various diseases, particularly in the field of neurology in neurodegenerative diseases such as Parkinson’s disease, as well as in stroke and chronic neuroinflammatory diseases such as multiple sclerosis (MS). However, our understanding of how biomarker concentrations are affected by normal aging processes, and renal function remains limited, which makes the interpretation of biomarker concentrations at the single-subject level and the definition of cut-off values for pathological conditions difficult. While the influence of chronic kidney disease (CKD) on some biomarkers has been well studied, less is known about the behavior of different biomarkers in the context of non-impaired renal function.

So far, neurofilament light chain (NfL) as a biomarker for neuroaxonal damage is one of the most intensively studied biomarker in neurological diseases. Progress has been made regarding the pathophysiology and significance of NfL level changes in the context of different diseases, and insights have been gained on the effects of aging processes, body weight, or impaired renal function on serum concentrations [1]. While studies mainly reported increasing NfL levels with aging [2,3,4], there have been inconclusive reports on the influence of impaired renal function. It has been hypothesized that NfL is excreted via the kidneys, so impaired renal function should be associated with decreased renal excretion of NfL and, thus, higher serum concentrations [5,6,7,8,9]. This hypothesis is supported by evidence that serum and urine NfL concentrations are strongly correlated in patients with good renal function [10]. Accordingly, numerous studies have reported that impaired renal function is associated with increasing serum concentrations of NfL [2,6,8,11]. Correlations between renal function parameters, including estimated glomerular filtration rate (eGFR), creatinine, and cystatin C with NfL in serum and cerebrospinal fluid (CSF) have been demonstrated [2,3,6,7,8,12], even after correction for age and gender [6,13]. However, age remains the strongest predictor of elevated NfL serum concentrations [3,8]. In a cohort of CKD patients, plasma NfL levels were higher than in patients with normal kidney function, while both groups showed no differences in CSF and MRI features [14]. Interestingly, Tang and colleagues demonstrated that NfL levels do not require adjustment for renal function when comparing them to other measures of neurodegeneration [7]. Only a few studies have failed to demonstrate correlations between creatinine or eGFR with serum NfL [15] or plasma and CSF NfL [7]. Thus, while there are numerous studies focusing on pathological kidney function, the relationship between individual fluctuations of kidney function within physiological ranges and serum NfL has not yet been sufficiently investigated.

Regarding other biomarkers, such as glial fibrillary acidic protein (GFAP), a marker of astroglial damage or activation [16], ubiquitin carboxy-terminal hydrolase L1 (UCH-L1), a marker for neuroaxonal integrity [17], and total TAU (tTAU), a marker for neurodegeneration [18], there have been only a few studies analyzing the impact of renal function on serum concentrations of these biomarkers. Various studies have shown, in line with the findings on NfL, an inverse correlation between eGFR and GFAP, suggesting that serum concentrations increase with impaired renal function [5,19]. In a different study, a moderate correlation between creatinine and tTAU was demonstrated, whereas this did not apply to GFAP or UCH-L1 [20]. Furthermore, no correlation between serum and urine concentrations has been found for GFAP and UCH-L1, indicating impaired filtration, e.g., due to molecular charge in the glomerulus in intact kidneys or tubular reabsorption [10,21]. For tTAU, an association between CKD and higher plasma levels of soluble tau has been described [22,23]. The highest correlation between urine and serum concentrations, with even higher concentrations in urine than in serum, has been reported for tTAU [10]. This has been hypothesized to be due to the hydrophilic structure of the unphosphorylated form, which facilitates the filtration in the renal glomerulus [24].

The current literature suggests that impaired renal function may affect serum and CSF concentrations of biomarkers by impacting blood–brain barrier integrity and clearance pathways. While these biomarkers, especially NfL, are well studied in renal disease, their behavior in the context of normal, physiological renal function is less well understood. To the best of our knowledge, there is a lack of studies investigating this association and comparing different biomarkers for serum and CSF values. Understanding the kinetics of these biomarkers within physiological ranges of kidney function could improve the interpretation of their concentrations and aid in developing more precise diagnostic models. Thus, an important question is whether adjustment for renal function is necessary even when renal function remains within physiological limits. Clinically relevant is whether adjusting standardized z-scores solely for the presence or absence of renal dysfunction is sufficient, or if all specific values (e.g., low-normal vs. high-normal values) should be corrected, for example, based on eGFR or creatinine stratified by deciles. Accordingly, the aim of this study was to evaluate the influence of varying renal function levels within the non-impaired creatinine clearance range on NfL, GFAP, UCH-L1, and tTAU levels in serum, as well as CSF, of patients with neuroimmunological diseases, especially MS, and other neurological diseases, as well as in somatoform controls.

## 2. Results

### 2.1. Patient Characteristics

In this study, which included n = 183 patients, the mean age was 37.07 years (SD 12.25), and 68.7% of the patients were female. The patients had an average BMI of 25.11 kg/m^2^ (SD 5.94). The mean creatinine concentration was 0.68 mg/dL (SD 0.16), with a mean eGFR of 114.43 mL/min/1.73 m^2^ (SD 16.16).

A small subgroup with impaired kidney function (n = 16), i.e., CKD-Epi eGFR under 90 mL/min/1.73 m^2^, was identified. In this subgroup, the average age was significantly higher (56.19 years, SD 13.91, *p* < 0.01) compared to the normal renal function group, and there was a proportion of only 37.5% female patients (*p* = 0.05). The mean creatinine concentration was 0.95 mg/dL (SD 0.12), and the mean BMI was 27.77 kg/m^2^ (SD 6.18) in the impaired kidney function cohort. Descriptive statistics for both cohorts and the respective biomarker concentrations are displayed in Table 1.

### 2.2. Relationship Between Biomarker Concentrations and Kidney Function

**Figure 1:**
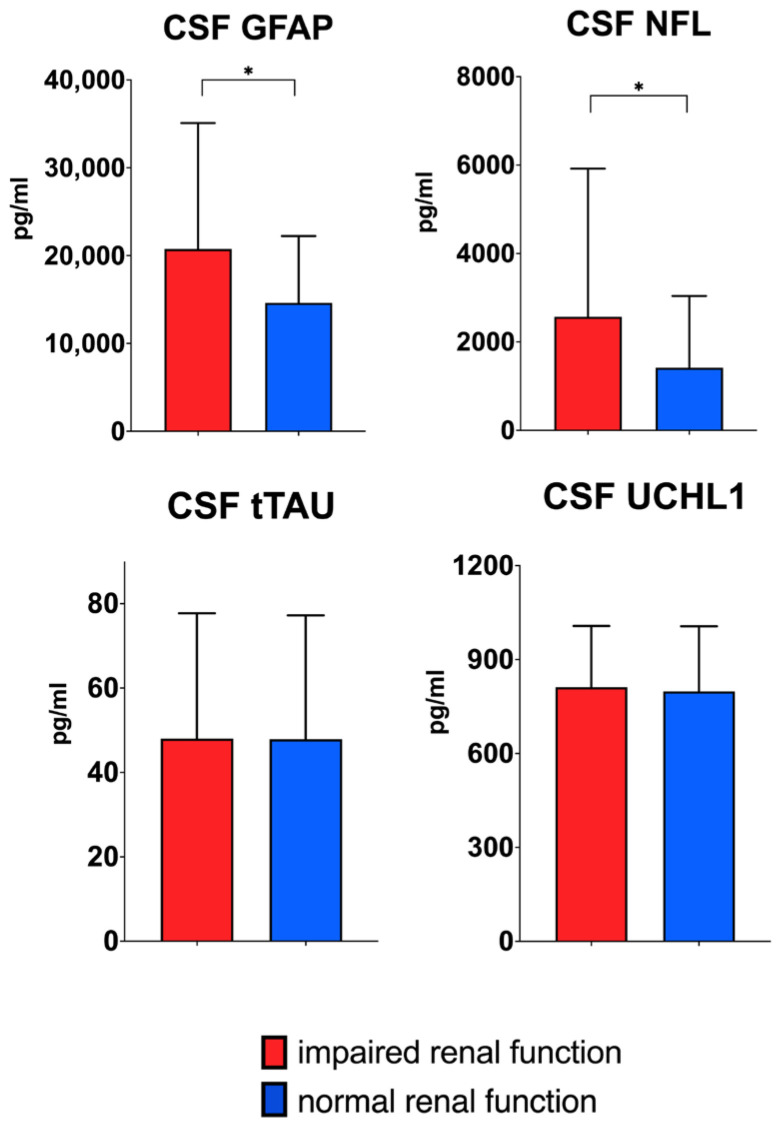
Boxplots displaying median values and interquartile range (IQR) of biomarker concentrations in cerebrospinal fluid (CSF) for normal and impaired renal function (defined as an eGFR under 90 mL/min/1.73 m^2^). For clarity, absolute values are displayed in this figure. For the *t*-test, log-transformed values were used. CSF NfL (*p* = 0.036) and CSF GFAP (*p* = 0.026) concentrations were higher in patients with impaired kidney function. * *p <* 0.05.

**Figure 2:**
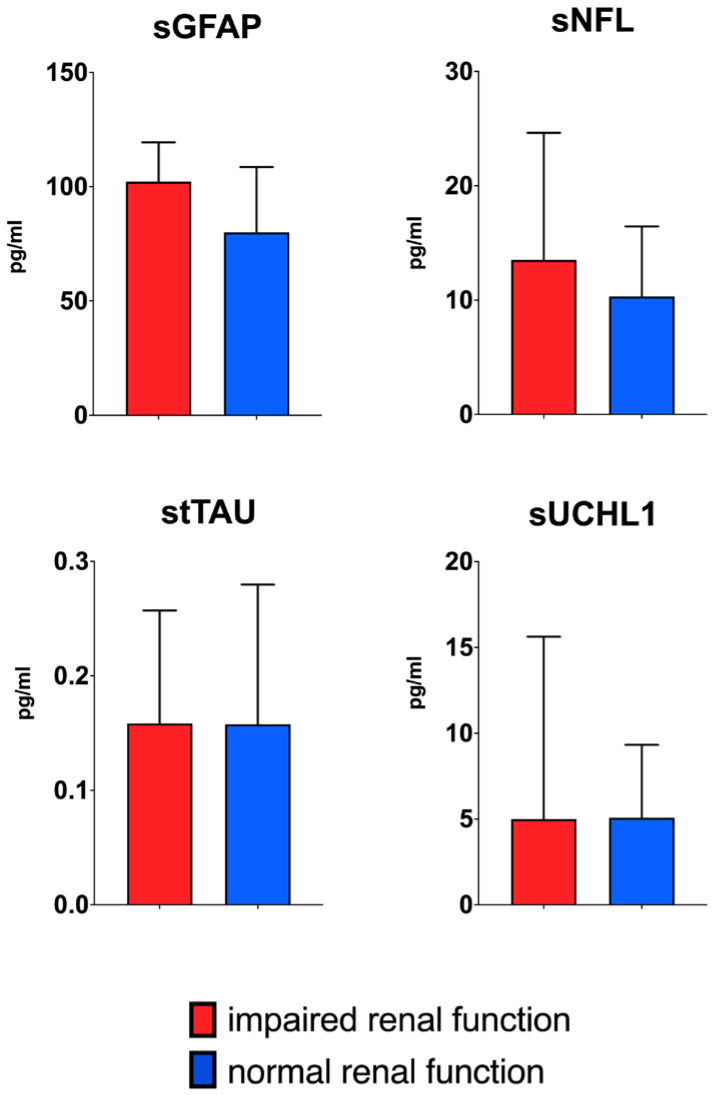
Boxplots showing median values and interquartile range (IQR) of biomarker concentrations in serum for normal and impaired renal function (defined as an eGFR under 90 mL/min/1.73 m^2^). For clarity, absolute values are displayed in this figure. Following log-transformation, no significant differences were found between the two groups for any of the biomarkers.

Following log-transformation of serum and CSF values, the analysis revealed that patients with impaired renal function exhibited higher CSF NfL (*p* = 0.036) and CSF GFAP (*p* = 0.026) concentrations than patients with normal renal function (Figure 1). However, when adjusting for BMI and age these differences did not remain significant. No significant difference between the two groups was found for the serum values of all four biomarkers (Figure 2).

**Figure 3:**
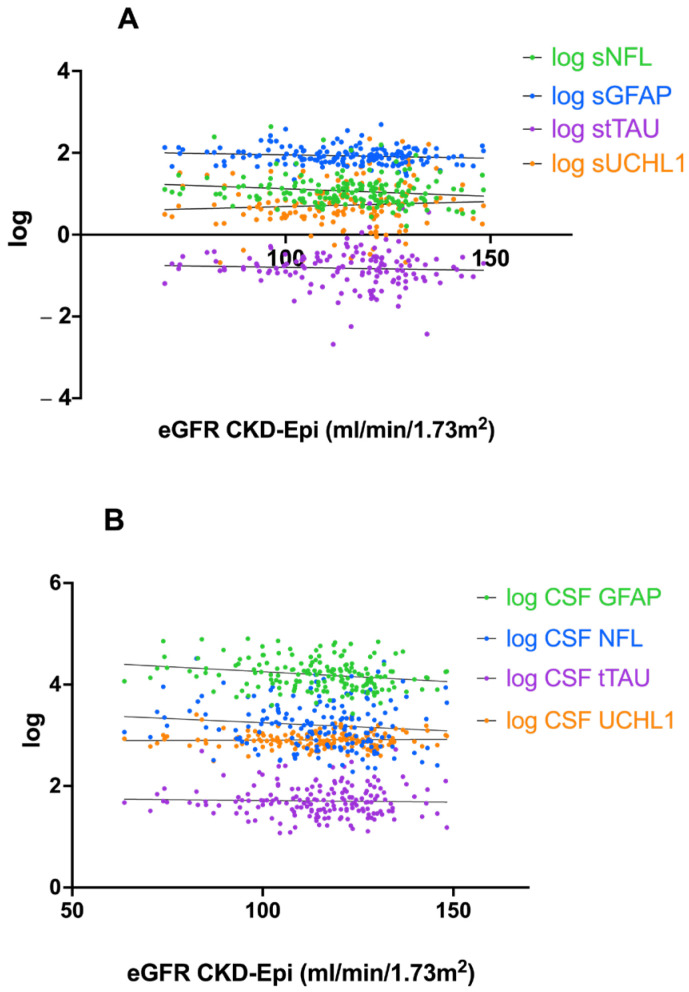
Scatterplots showing the association between kidney function, measured as eGFR CKD-Epi (ml/min/1.73 m^2^), and the log-transformed serum (**A**) or cerebrospinal fluid (CSF) concentrations (**B**) with the fitting of a linear regression curve. sNfL (*p* = 0.032), sGFAP (*p* = 0.097), sUCHL1 (*p* = 0.371), stTAU (*p* = 0.548) cNfL (*p* = 0.137), cGFAP (*p* = 0.004), cUCHL1 (*p* = 0.718), ctTAU (*p* = 0.644).

eGFR correlated negatively with log-transformed CSF GFAP (r = −0.217, *p* = 0.004) and serum NfL (r = −0.164, *p* = 0.032). However, after adjustment for BMI and age these correlations did not remain significant. For UCHL1 and tTAU, there were no significant correlations detected for eGFR, either in serum or CSF concentrations. A linear regression of the log-transformed serum and CSF concentrations with eGFR is displayed in Figure 3A,B. According to the above-mentioned correlations, only serum NfL (*p* = 0.032) and CSF GFAP (*p* = 0.004) were statistically significant.

Additionally, a subgroup analysis for patients with MS phenotype (n = 117) was performed as a representative example. In this subgroup, after adjusting for age and BMI, only the correlation between UCHL1 in CSF and eGFR showed a significant but weak correlation (ρ = 0.218, *p* = 0.029). For all other biomarkers, no significant correlations were observed between renal function and the log-transformed biomarker concentrations in serum and CSF (sNfL (ρ = 0.099, *p* = 0.324), sGFAP (ρ = 0.020, *p* = 0.840), sUCHL1 (ρ = 0.086, *p* = 0.418), stTAU (ρ = 0.027, *p* = 0.806), cNfL (ρ = 0.090, *p* = 0.370), cGFAP (ρ = 0.004, *p* = 0.969), and cTAU (ρ = 0.149, *p* = 0.137)).

Importantly, when excluding all patients with impaired renal function, only CSF GFAP showed a negative correlation with eGFR (−0.159, *p* = 0.046), which did not remain significant after controlling for age and BMI. No significant results for the correlation between eGFR and the other four biomarkers were found in the dataset focusing solely on patients with normal renal function (sNfL (ρ = −0.123, *p* = 0.125), sGFAP (ρ = −0.145, *p* = 0.071), sUCHL1 (ρ = 0.154, *p* = 0.074), stTAU (ρ = 0.085, *p* = 0.336), cNfL (ρ = 0.100, *p* = 0.209), cUCHL1 (ρ = 0.106, *p* = 0.184), cTAU (ρ = 0.056, *p* = 0.483)).

## 3. Discussion

The main aim of this study was to evaluate the effects of variations in renal function within physiological ranges on serum and CSF biomarker levels. We found no correlation between eGFR and the biomarker levels in the cohort with normal renal function; also, the correlation with CSF GFAP did not remain significant after controlling for age and BMI. This suggests that normal fluctuations within the physiological range of renal function have no significant impact on the concentrations of NfL, GFAP, UCHL1, and tTAU in serum or CSF. Therefore, we suggest that in the absence of explicit renal impairment and with eGFR not exceeding pathological cut-off values, the serum and CSF concentrations of these biomarkers can be reliably interpreted without the need for adjustments based on the individual level of kidney function.

Furthermore, we demonstrated a negative correlation between GFR and both CSF GFAP and serum NfL, indicating that impaired renal function might be associated with increased levels of these biomarkers. These findings are in line with the majority of studies [2,6,8]. However, after adjusting for age and BMI, the correlations were no longer significant, which aligns with the well-established understanding that age is the main factor influencing NfL concentrations [6,13] and supports the recommended use of z-scores for interpretation [25]. In contrast, our results are not consistent with previous studies that have reported correlations between eGFR and serum GFAP [5,19] or CSF NfL [7]. A potential explanation for this discrepancy could be the limited number of patients with pathological renal function in our cohort compared to other studies.

Interestingly, patients with impaired renal function exhibited significantly higher CSF concentrations of NfL and GFAP compared to those with normal renal function. This could indicate that renal impairment is linked to an increase in these biomarkers in the CSF, aligning with previous research [7]. Nevertheless, once BMI and age were considered, these differences lost statistical significance, further emphasizing the importance of adjusting for these confounders when interpreting biomarker levels. Pathophysiologically, the question arises as to whether impaired renal function also affects the blood–brain barrier due to uremic processes and whether this can explain the higher CSF concentrations. Mair and colleagues showed in an experimental mouse model of renal failure that the blood–brain barrier remains functional but cannot prevent the accumulation of uremic solutes in the CSF [26]. Further investigations in this area are required to fully understand the underlying pathophysiological processes between renal function and the blood–brain barrier. Neurological patients with highly impaired renal function might exhibit abnormally high biomarker CSF levels.

In our study, no significant correlations were found between eGFR and either UCHL1 or tTAU in both serum and CSF samples, suggesting that these particular biomarkers may not be directly influenced by renal function. This finding for UCHL1 aligns with previous work [10,20] but is contrary to the literature regarding the correlation between renal function and tTAU [20,23].

One main limitation of this study, as mentioned above, is the small sample size of subjects with impaired renal function, which limits the comparability between the two subgroups and the interpretation of the results in the exploratory analysis. Nevertheless, the focus of our study was primarily on patients with normal kidney function. Pathological kidney function has already been extensively examined in numerous prior investigations. We only evaluated the parameters eGFR and creatinine; therefore, the influence of other relevant metabolic parameters, like cystatin C or albuminuria, remains unclear. Given that the primary focus of this study is on physiological kidney function, significant differences in the results for other parameters are not expected. Moreover, we did not control for other diseases, e.g., diabetes mellitus, or for medication use. Another limitation to mention is the cross-sectional study design. Long-term follow-ups would have been helpful to validate these findings by assessing the stability of these correlations over time and providing a clearer understanding of the dynamics between renal function and biomarker concentrations. It should also be noted that our cohort is heterogeneous, consisting primarily of patients with neuroimmunological diseases, as well as other neurological conditions and somatoform disorders. However, since these biomarkers are not specific to any single disease, their interpretation across a variety of neurological conditions and in subjects without neurological disease (somatoform controls) is highly relevant. This diversity within the cohort can therefore be advantageous in addressing the objective of our study. We therefore added an exploratory subgroup analysis of the MS phenotype patients, which revealed similar results.

To conclude, our results suggest that serum and CSF concentrations of NfL, GFAP, tTAU, and UCHL1 are not significantly affected by fluctuations in renal function within the normal physiological range. Nevertheless, in patients with compromised renal function, comorbidities and reduced kidney clearance may particularly influence NfL and GFAP levels and should be considered when interpreting these concentrations. Even in the absence of kidney impairment, it remains crucial to consider other confounding variables, such as age and BMI, as established in multiple studies. This emphasizes the complexity of interpreting biomarker data in the context of confounding pathological conditions and underscores the need for larger, more comprehensive studies to further explore these relationships.

## 4. Methods and Materials

### 4.1. Study Design and Biomarker Measurement

In this observational, single-center cohort study, we recruited n = 183 patients (MS n = 87, radiological isolated syndrome (RIS) and clinically isolated syndrome (CIS) n = 30, neuromyelitis optica spectrum disease (NMOSD) and myelin oligodendrocyte glycoprotein antibody disease (MOGAD) n = 4, other neurological diseases n = 39, somatoform controls n = 22) who underwent a lumbar puncture as part of a diagnostic check-up at the University Hospital Frankfurt. For the MS patients, the 2017 revision of the McDonald criteria was applied [27]. Patients classified as somatoform disease controls presented with subjective neurological symptoms; however, the diagnostic workup revealed no underlying organic condition.

Serum samples were either drawn from the remnants of a routine blood collection (S-Monovette, 4.7 mL; Sarstedt AG & Co. KG, Nümbrecht, Germany) or collected via venous puncture. Serum and CSF samples for biomarker analysis were collected during the clinically scheduled sample collection and before any corticosteroid therapy. Creatinine in serum, as well as the eGFR, specifically, the Chronic Kidney Disease Epidemiology Collaboration (CKD-Epi) formula [28], was assessed to evaluate kidney function. Impaired kidney function was defined as a CKD-Epi eGFR under 90 mL/min/1.73 m^2^.

For the measurement of GFAP, UCHL1 and tTAU concentrations in serum and CSF, we employed the single molecule array HD-1 analyzer (SIMOA; Quanterix, Boston, MA, USA) using the Neurology 4-Plex A Advantage Kit (Quanterix, Boston, MA, USA). The sample acquisition and measurement protocols have been reported in previous works by our group [29,30,31,32].

### 4.2. Statistical Analysis

Statistical analyses were performed using IBM SPSS^®^ Statistics, Version 29 (Statistical Package for the Social Sciences), and Graph Pad Prism (Version 10.2.3.). Descriptive statistics were used to present baseline characteristics of both cohorts, including median and interquartile ranges (IQRs) of the biomarker concentrations. Group differences between impaired and non-impaired kidney function were assessed using *t*-tests for independent samples or the Mann–Whitney U test for parametric/non-parametric data. For the biomarker concentrations, we employed log-transformed values for the *t*-tests. Similarly, for correlational analysis, serum and CSF biomarker levels were log-transformed. Pearson correlation was used to evaluate the associations between eGFR and log-transformed biomarker levels. In a second step, we adjusted the results for the covariates age and body mass index (BMI) by applying partial correlational analysis. The relationship between eGFR and serum/CSF biomarker levels was displayed in scatterplots with a fitted linear regression curve. For all analyses, the level of significance was set to *p <* 0.05.

### 4.3. Standard Protocol Approvals and Patient Consents

This study was performed in accordance with The Code of Ethics of the World Medical Association (Declaration of Helsinki) for experiments involving humans and was approved by the local ethics committee at the University Hospital Frankfurt (number 110/11 on 23 August 2019 and number 173/19 on 23 April 2019). It followed the guidelines for reporting observational studies (STROBE) [33]. Written informed consent was obtained from all patients.

## Figures and Tables

**Table 1:** Descriptive statistics of the whole cohort, split according to renal function.

	Whole Cohort	Normal Kidney Function (eGFR > 90 mL/min/1.73 m^2^)	Impaired Kidney Function (eGFR < 90 mL/min/1.73 m^2^)	
**N (%)**	183 (100)	167 (91.25)	16 (8.74)	
**Age (mean, SD)**	37.07 (12.25)	35.24 (10.41)	56.19 (13.91)	*p* < 0.01
**Sex (female, %)**	125 (68.7)	119 (71.3)	6 (37.5)	*p* = 0.05
**BMI kg/m^2^ (mean, SD)**	25.11 (5.94)	24.87 (5.88)	27.77 (6.18)	n.s.
**Creatinine (mg/dL, mean, SD)**	0.68 (0.16)	0.65 (0.14)	0.95 (0.12)	
**eGFR CKD-Epi (mL/min/1.73 m^2^, mean, SD)**	114.43 (16.16)	117.71 (12.54)	80.31 (7.77)	
**sGFAP pg/mL (median, IQR)**	80.45 (48.05)	79.98 (47.92)	103.59 (77.45)	n.s.
**cGFAP pg/mL (median, IQR)**	15,326.80 (14,219.60)	14,594.42 (13,362.91)	21,048.68 (27,364.01)	*p* = 0.026
**sNfL pg/mL (median, IQR)**	10.57 (9.41)	10.14 (9.45)	13.16 (15.48)	n.s.
**cNfL pg/mL (median, IQR)**	1453.30 (2456.86)	1414.56 (2408.02)	2948.88 (6538.82)	*p* = 0.036
**stTAU pg/mL (median, IQR)**	0.16 (0.18)	0.15 (0.19)	0.17 (0.14)	n.s.
**ctTAU pg/mL (median, IQR)**	47.78 (45.89)	47.58 (46.34)	49.79 (42.54)	n.s.
**sUCHL1 pg/mL (median, IQR)**	5.01 (6.86)	5.05 (6.34)	4.06 (13.75)	n.s.
**cUCHL1 pg/mL (median, IQR)**	798.61 (365.02)	796.12 (364.90)	821.11 (601.65)	n.s.

For the biomarker concentrations, log-transformed serum and CSF values were analyzed using a *t*-test for independent samples. BMI = body mass index, CKD-Epi = Chronic Kidney Disease Epidemiology Collaboration, eGFR = estimated glomerular filtration rate, GFAP = glial fibrillary acidic protein, IQR = interquartile range, NfL = neurofilament light chain, n.s. = non-significant, SD = standard deviation, tTAU = total tau, UCHL1 = ubiquitin C-terminal hydrolase L1.

## Data Availability

Anonymized data not published within this article will be made available upon a reasonable request from any qualified investigator.

## Abbreviations

BBB	Blood–brain barrier
BMI	Body mass index
CIS	Clinically isolated syndrome
CKD	Chronic kidney disease
CKD-Epi	Chronic Kidney Disease Epidemiology Collaboration
CSF	Cerebrospinal fluid
EDSS	Expanded Disability Status Scale
eGFR	Estimated glomerular filtration rate
GFAP	Glial fibrillary acidic protein
IQR	Interquartile range
MOGAD	Myelin oligodendrocyte glycoprotein antibody disease
MS	Multiple sclerosis
NfL	Neurofilament light chain
NID	Neuroinflammatory disease
NMOSD	Neuromyelitis optica spectrum disease
OCBs	Oligoclonal bands
PMS	Progressive multiple sclerosis
RIS	Radiologically isolated syndrome
ROC	Receiver operating characteristic
RRMS	Relapsing-remitting multiple sclerosis
SD	Standard deviation
SIMOA	Single molecule array
tTAU	Total tau
UCHL1	Ubiquitin C-terminal hydrolase L1
